# Tetra­aqua­bis[2-(thio­semicarbazonometh­yl)benzene­sulfonato]calcium(II)

**DOI:** 10.1107/S1600536809035971

**Published:** 2009-09-12

**Authors:** Zhang Wei, Chen Yuan-Tao

**Affiliations:** a Department of Chemistry, Qinghai Normal University, Xining 810008, People’s Republic of China

## Abstract

In the title compound, [Ca(C_8_H_8_N_3_O_3_S_2_)_2_(H_2_O)_4_], the Ca atom (site symmetry 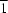) adopts a slightly distorted octa­hedral CaO_6_ geometry and the mol­ecular conformation is stabilized by intra­molecular N—H⋯N inter­actions. In the crystal, the mol­ecules are linked by O—H⋯O, O—H⋯S, N—H⋯O and N—H⋯S hydrogen bonds.

## Related literature

For background to Schiff bases, see: Sawant *et al.* (2009 ▶).
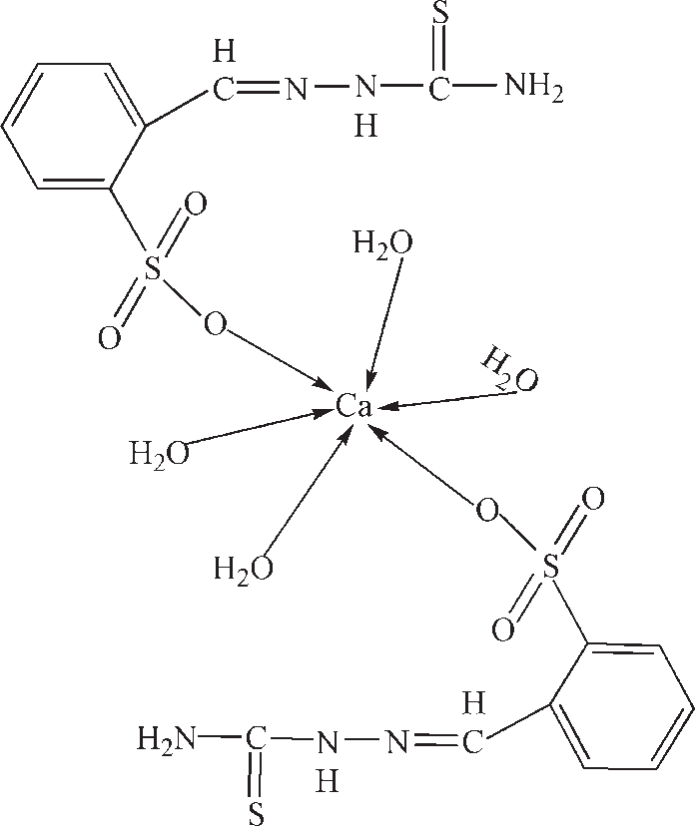


## Experimental

### 

#### Crystal data

[Ca(C_8_H_8_N_3_O_3_S_2_)_2_(H_2_O)_4_]
*M* *_r_* = 628.73
Triclinic, 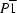

*a* = 6.9123 (11) Å
*b* = 9.6383 (13) Å
*c* = 10.9481 (17) Å
α = 64.372 (1)°
β = 87.708 (2)°
γ = 83.225 (2)°
*V* = 652.99 (17) Å^3^
*Z* = 1
Mo *K*α radiation
μ = 0.62 mm^−1^
*T* = 298 K
0.31 × 0.15 × 0.12 mm

#### Data collection

Bruker SMART CCD diffractometer
Absorption correction: multi-scan *SADABS* (Bruker, 2000 ▶) *T*_min_ = 0.831, *T*_max_ = 0.929
2223 measured reflections
2223 independent reflections
1781 reflections with *I* > 2σ(*I*)

#### Refinement

*R*[*F* ^2^ > 2σ(*F* ^2^)] = 0.078
*wR*(*F* ^2^) = 0.227
*S* = 1.04
2223 reflections
170 parameters
H-atom parameters constrained
Δρ_max_ = 0.60 e Å^−3^
Δρ_min_ = −0.55 e Å^−3^

### 

Data collection: *SMART* (Bruker, 2000 ▶); cell refinement: *SAINT* (Bruker, 2000 ▶); data reduction: *SAINT*; program(s) used to solve structure: *SHELXS97* (Sheldrick, 2008 ▶); program(s) used to refine structure: *SHELXL97* (Sheldrick, 2008 ▶); molecular graphics: *SHELXTL* (Sheldrick, 2008 ▶); software used to prepare material for publication: *SHELXTL*.

## Supplementary Material

Crystal structure: contains datablocks global, I. DOI: 10.1107/S1600536809035971/hb5075sup1.cif

Structure factors: contains datablocks I. DOI: 10.1107/S1600536809035971/hb5075Isup2.hkl

Additional supplementary materials:  crystallographic information; 3D view; checkCIF report

## Figures and Tables

**Table 1:** Selected bond lengths (Å)

Ca1—O4	2.310 (4)
Ca1—O5	2.313 (6)
Ca1—O1	2.362 (4)

**Table 2:** Hydrogen-bond geometry (Å, °)

*D*—H⋯*A*	*D*—H	H⋯*A*	*D*⋯*A*	*D*—H⋯*A*
N3—H3*A*⋯N2	0.86	2.28	2.636 (7)	105
O5—H5*C*⋯O2^i^	0.85	2.07	2.840 (9)	150
N1—H1⋯S2^ii^	0.86	2.60	3.441 (6)	166
N3—H3*B*⋯O2^iii^	0.86	2.34	3.035 (7)	138
O4—H4*C*⋯S2^iv^	0.85	2.42	3.261 (6)	173
O4—H4*D*⋯O3^v^	0.85	1.87	2.712 (8)	171
O5—H5*D*⋯S2^ii^	0.85	2.42	3.197 (8)	152

Symmetry codes: (i) 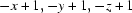; (ii) 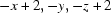; (iii) 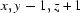; (iv) 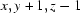; (v) 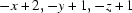.

**Fig. 1.**
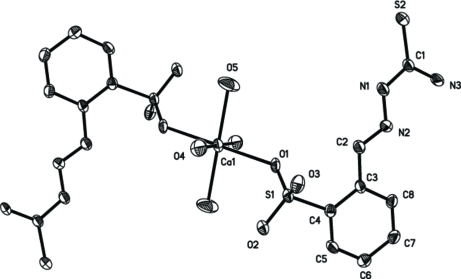
The molecular structure of (I) showing 30% displacement ellipsoids. Unlabelled atoms are generated by the symmetry operation (1–x, 1–y, 1–z).

## supplementary crystallographic information

### Comment

Schiff base metal complexes have been of interest in coordination chemistry for many years due to their facile synthesis, strong coordination function and wide applications (*e.g.* Sawant, *et al.*, 2009). Ca complexes with Schiff base ligand have received little attention. In this paper, we report on the synthesis and crystal structure of the title compound, (I), (Scheme I).

The Ca(II) center is Six-coordinate with two O donors of 2-formyl-benzenesulfonate-thiosemicarbazide ligands and four O donors of coordinated water molecules, and adopts distorted octahedral coordination. The bond distances of Ca—O are in the range of 2.310 (4)–2.362 (4), which are consistent with the bond lengths reported previously. In the crystal packing, the molecules form a one-dimensional chain structure by the interaction of hydrogen bonds.

### Experimental

A solution of 1.0 mmol 2-formyl-benzenesulfonate-thiosemicarbazide was added to a solution of 0.5 mmol Ca(ClO_4_)_2_.4H_2_O in 5 ml e thanol at room temperature. The mixture was refluxed for 4 h with stirring, then the resulting precipitate was filtered, washed, and dried *in vacuo* over P_4_O_10_ for 48 h. Colourless blocks of (I) were obtained by slowly evaporating from methanol at room temperature.

### Crystal data

**Table:**

[Ca(C_8_H_8_N_3_O_3_S_2_)_2_(H_2_O)_4_]	*Z* = 1
*M**_r_* = 628.73	*F*(000) = 326
Triclinic, *P*1	*D*_x_ = 1.599 Mg m^−^^3^
Hall symbol: -P 1	Mo *K*α radiation, λ = 0.71073 Å
*a* = 6.9123 (11) Å	Cell parameters from 1563 reflections
*b* = 9.6383 (13) Å	θ = 3.6–27.6°
*c* = 10.9481 (17) Å	µ = 0.62 mm^−^^1^
α = 64.372 (1)°	*T* = 298 K
β = 87.708 (2)°	Block, colourless
γ = 83.225 (2)°	0.31 × 0.15 × 0.12 mm
*V* = 652.99 (17) Å^3^	

### Data collection

**Table:**

Bruker SMART CCD diffractometer	2223 independent reflections
Radiation source: fine-focus sealed tube	1781 reflections with *I* > 2σ(*I*)
graphite	*R*_int_ = 0.0000
ω scans	θ_max_ = 25.0°, θ_min_ = 2.1°
Absorption correction: multi-scan *SADABS* (Bruker, 2000)	*h* = −8→8
*T*_min_ = 0.831, *T*_max_ = 0.929	*k* = −10→11
2223 measured reflections	*l* = −9→13

### Refinement

**Table:**

Refinement on *F*^2^	Primary atom site location: structure-invariant direct methods
Least-squares matrix: full	Secondary atom site location: difference Fourier map
*R*[*F*^2^ > 2σ(*F*^2^)] = 0.078	Hydrogen site location: inferred from neighbouring sites
*wR*(*F*^2^) = 0.227	H-atom parameters constrained
*S* = 1.03	*w* = 1/[σ^2^(*F*_o_^2^) + (0.1501*P*)^2^ + 0.6556*P*] where *P* = (*F*_o_^2^ + 2*F*_c_^2^)/3
2223 reflections	(Δ/σ)_max_ < 0.001
170 parameters	Δρ_max_ = 0.60 e Å^−^^3^
0 restraints	Δρ_min_ = −0.55 e Å^−^^3^

### Special details

**Table:**

Geometry. All e.s.d.'s (except the e.s.d. in the dihedral angle between two l.s. planes) are estimated using the full covariance matrix. The cell e.s.d.'s are taken into account individually in the estimation of e.s.d.'s in distances, angles and torsion angles; correlations between e.s.d.'s in cell parameters are only used when they are defined by crystal symmetry. An approximate (isotropic) treatment of cell e.s.d.'s is used for estimating e.s.d.'s involving l.s. planes.
Refinement. Refinement of *F*^2^ against ALL reflections. The weighted *R*-factor *wR* and goodness of fit *S* are based on *F*^2^, conventional *R*-factors *R* are based on *F*, with *F* set to zero for negative *F*^2^. The threshold expression of *F*^2^ > σ(*F*^2^) is used only for calculating *R*-factors(gt) *etc*. and is not relevant to the choice of reflections for refinement. *R*-factors based on *F*^2^ are statistically about twice as large as those based on *F*, and *R*- factors based on ALL data will be even larger.

### Fractional atomic coordinates and isotropic or equivalent isotropic displacement parameters (Å^2^)

**Table:**

	*x*	*y*	*z*	*U*_iso_*/*U*_eq_	
Ca1	0.5000	0.5000	0.5000	0.0345 (5)	
S1	0.7458 (2)	0.67806 (16)	0.65974 (13)	0.0354 (4)	
S2	1.0140 (3)	−0.13332 (17)	1.21326 (16)	0.0449 (5)	
O1	0.6451 (7)	0.5503 (5)	0.6662 (4)	0.0438 (11)	
O2	0.6760 (7)	0.8251 (5)	0.5486 (4)	0.0501 (12)	
O3	0.9554 (7)	0.6437 (6)	0.6624 (5)	0.0507 (12)	
O4	0.7857 (7)	0.5634 (6)	0.3822 (5)	0.0556 (13)	
H4C	0.8364	0.6474	0.3401	0.083*	
H4D	0.8592	0.4914	0.3730	0.083*	
O5	0.6194 (10)	0.2408 (6)	0.5851 (8)	0.096 (3)	
H5C	0.5528	0.1878	0.5611	0.143*	
H5D	0.7093	0.1806	0.6394	0.143*	
N1	0.9136 (7)	0.1670 (6)	1.0860 (5)	0.0354 (11)	
H1	0.9541	0.1587	1.0139	0.043*	
N2	0.8339 (7)	0.3076 (5)	1.0789 (5)	0.0337 (11)	
N3	0.8802 (9)	0.0645 (6)	1.3157 (5)	0.0506 (14)	
H3A	0.8420	0.1565	1.3071	0.061*	
H3B	0.8874	−0.0123	1.3947	0.061*	
C1	0.9278 (8)	0.0419 (6)	1.2074 (6)	0.0353 (13)	
C2	0.8137 (8)	0.4179 (6)	0.9586 (6)	0.0352 (12)	
H2	0.8529	0.4001	0.8838	0.042*	
C3	0.7269 (8)	0.5739 (6)	0.9407 (5)	0.0309 (12)	
C4	0.6848 (8)	0.6971 (6)	0.8118 (5)	0.0311 (12)	
C5	0.6039 (9)	0.8407 (7)	0.7991 (6)	0.0390 (13)	
H5	0.5791	0.9211	0.7133	0.047*	
C6	0.5592 (9)	0.8668 (7)	0.9128 (7)	0.0429 (14)	
H6	0.5031	0.9635	0.9034	0.052*	
C7	0.5998 (9)	0.7453 (8)	1.0420 (7)	0.0441 (15)	
H7	0.5721	0.7615	1.1190	0.053*	
C8	0.6807 (9)	0.6020 (7)	1.0545 (6)	0.0369 (13)	
H8	0.7054	0.5219	1.1406	0.044*	

### Atomic displacement parameters (Å^2^)

**Table:**

	*U*^11^	*U*^22^	*U*^33^	*U*^12^	*U*^13^	*U*^23^
Ca1	0.0392 (9)	0.0391 (9)	0.0220 (8)	−0.0068 (7)	−0.0025 (6)	−0.0094 (7)
S1	0.0473 (9)	0.0352 (8)	0.0200 (7)	−0.0087 (6)	−0.0001 (6)	−0.0074 (6)
S2	0.0613 (11)	0.0331 (8)	0.0317 (8)	−0.0016 (7)	−0.0043 (7)	−0.0066 (7)
O1	0.069 (3)	0.044 (2)	0.0195 (19)	−0.016 (2)	−0.0045 (18)	−0.0119 (18)
O2	0.080 (3)	0.040 (2)	0.023 (2)	−0.011 (2)	−0.005 (2)	−0.0059 (19)
O3	0.053 (3)	0.066 (3)	0.042 (3)	−0.011 (2)	0.008 (2)	−0.031 (2)
O4	0.056 (3)	0.058 (3)	0.053 (3)	−0.017 (2)	0.019 (2)	−0.023 (2)
O5	0.103 (5)	0.043 (3)	0.123 (6)	0.013 (3)	−0.073 (4)	−0.018 (3)
N1	0.042 (3)	0.034 (2)	0.022 (2)	0.001 (2)	−0.0021 (19)	−0.005 (2)
N2	0.038 (3)	0.030 (2)	0.029 (3)	−0.0019 (19)	−0.0006 (19)	−0.009 (2)
N3	0.082 (4)	0.034 (3)	0.024 (3)	0.000 (3)	0.000 (2)	−0.004 (2)
C1	0.040 (3)	0.035 (3)	0.025 (3)	−0.007 (2)	−0.003 (2)	−0.006 (2)
C2	0.039 (3)	0.034 (3)	0.027 (3)	−0.004 (2)	−0.004 (2)	−0.008 (2)
C3	0.032 (3)	0.031 (3)	0.026 (3)	−0.008 (2)	−0.005 (2)	−0.007 (2)
C4	0.032 (3)	0.033 (3)	0.025 (3)	−0.010 (2)	0.002 (2)	−0.008 (2)
C5	0.045 (3)	0.031 (3)	0.032 (3)	−0.005 (2)	−0.003 (2)	−0.005 (2)
C6	0.047 (3)	0.040 (3)	0.045 (4)	0.000 (3)	0.000 (3)	−0.021 (3)
C7	0.055 (4)	0.047 (3)	0.037 (3)	−0.013 (3)	0.007 (3)	−0.023 (3)
C8	0.045 (3)	0.037 (3)	0.028 (3)	−0.008 (2)	−0.001 (2)	−0.012 (2)

### Geometric parameters (Å, °)

**Table:**

Ca1—O4^i^	2.310 (4)	N1—H1	0.8600
Ca1—O4	2.310 (4)	N2—C2	1.286 (7)
Ca1—O5	2.313 (6)	N3—C1	1.318 (8)
Ca1—O5^i^	2.313 (6)	N3—H3A	0.8599
Ca1—O1	2.362 (4)	N3—H3B	0.8599
Ca1—O1^i^	2.362 (4)	C2—C3	1.484 (8)
S1—O3	1.446 (5)	C2—H2	0.9300
S1—O2	1.455 (4)	C3—C8	1.403 (8)
S1—O1	1.459 (4)	C3—C4	1.410 (8)
S1—C4	1.781 (6)	C4—C5	1.380 (9)
S2—C1	1.698 (6)	C5—C6	1.389 (9)
O4—H4C	0.8497	C5—H5	0.9300
O4—H4D	0.8503	C6—C7	1.405 (10)
O5—H5C	0.8504	C6—H6	0.9300
O5—H5D	0.8499	C7—C8	1.378 (9)
N1—C1	1.351 (7)	C7—H7	0.9300
N1—N2	1.372 (7)	C8—H8	0.9300
			
O4^i^—Ca1—O4	180.0	H5C—O5—H5D	108.9
O4^i^—Ca1—O5	90.5 (2)	C1—N1—N2	119.3 (5)
O4—Ca1—O5	89.5 (2)	C1—N1—H1	120.4
O4^i^—Ca1—O5^i^	89.5 (2)	N2—N1—H1	120.3
O4—Ca1—O5^i^	90.5 (2)	C2—N2—N1	115.2 (5)
O5—Ca1—O5^i^	180.0	C1—N3—H3A	119.7
O4^i^—Ca1—O1	94.41 (17)	C1—N3—H3B	120.2
O4—Ca1—O1	85.59 (17)	H3A—N3—H3B	120.0
O5—Ca1—O1	96.42 (19)	N3—C1—N1	117.5 (5)
O5^i^—Ca1—O1	83.58 (19)	N3—C1—S2	123.7 (4)
O4^i^—Ca1—O1^i^	85.59 (17)	N1—C1—S2	118.8 (5)
O4—Ca1—O1^i^	94.41 (17)	N2—C2—C3	119.1 (6)
O5—Ca1—O1^i^	83.58 (19)	N2—C2—H2	120.5
O5^i^—Ca1—O1^i^	96.42 (19)	C3—C2—H2	120.5
O1—Ca1—O1^i^	180.0	C8—C3—C4	117.7 (5)
O4^i^—Ca1—H5C	83.1	C8—C3—C2	119.9 (5)
O4—Ca1—H5C	96.9	C4—C3—C2	122.3 (5)
O5—Ca1—H5C	16.4	C5—C4—C3	120.7 (5)
O5^i^—Ca1—H5C	163.6	C5—C4—S1	117.3 (4)
O1—Ca1—H5C	111.4	C3—C4—S1	121.9 (4)
O1^i^—Ca1—H5C	68.6	C4—C5—C6	120.9 (6)
O3—S1—O2	113.0 (3)	C4—C5—H5	119.5
O3—S1—O1	112.4 (3)	C6—C5—H5	119.5
O2—S1—O1	112.7 (3)	C5—C6—C7	119.1 (6)
O3—S1—C4	106.2 (3)	C5—C6—H6	120.4
O2—S1—C4	106.4 (3)	C7—C6—H6	120.4
O1—S1—C4	105.5 (2)	C8—C7—C6	119.9 (6)
S1—O1—Ca1	133.1 (2)	C8—C7—H7	120.1
Ca1—O4—H4C	134.2	C6—C7—H7	120.1
Ca1—O4—H4D	117.6	C7—C8—C3	121.6 (6)
H4C—O4—H4D	108.2	C7—C8—H8	119.2
Ca1—O5—H5C	113.7	C3—C8—H8	119.2
Ca1—O5—H5D	137.2		
			
O3—S1—O1—Ca1	−98.8 (4)	C8—C3—C4—S1	177.3 (4)
O2—S1—O1—Ca1	30.3 (5)	C2—C3—C4—S1	−3.8 (7)
C4—S1—O1—Ca1	145.9 (3)	O3—S1—C4—C5	115.4 (5)
O4^i^—Ca1—O1—S1	−131.7 (4)	O2—S1—C4—C5	−5.2 (5)
O4—Ca1—O1—S1	48.3 (4)	O1—S1—C4—C5	−125.1 (5)
O5—Ca1—O1—S1	137.3 (4)	O3—S1—C4—C3	−61.0 (5)
O5^i^—Ca1—O1—S1	−42.7 (4)	O2—S1—C4—C3	178.4 (4)
O1^i^—Ca1—O1—S1	61 (12)	O1—S1—C4—C3	58.5 (5)
C1—N1—N2—C2	−175.4 (5)	C3—C4—C5—C6	−1.1 (9)
N2—N1—C1—N3	−6.2 (8)	S1—C4—C5—C6	−177.5 (4)
N2—N1—C1—S2	176.3 (4)	C4—C5—C6—C7	0.9 (9)
N1—N2—C2—C3	179.4 (5)	C5—C6—C7—C8	−0.8 (9)
N2—C2—C3—C8	4.4 (8)	C6—C7—C8—C3	0.9 (9)
N2—C2—C3—C4	−174.4 (5)	C4—C3—C8—C7	−1.0 (8)
C8—C3—C4—C5	1.1 (8)	C2—C3—C8—C7	−179.9 (5)
C2—C3—C4—C5	180.0 (5)		

Symmetry codes: (i) −*x*+1, −*y*+1, −*z*+1.

### Hydrogen-bond geometry (Å, °)

**Table:**

*D*—H···*A*	*D*—H	H···*A*	*D*···*A*	*D*—H···*A*
N3—H3A···N2	0.86	2.28	2.636 (7)	105
O5—H5C···O2^i^	0.85	2.07	2.840 (9)	150
N1—H1···S2^ii^	0.86	2.60	3.441 (6)	166
N3—H3B···O2^iii^	0.86	2.34	3.035 (7)	138
O4—H4C···S2^iv^	0.85	2.42	3.261 (6)	173
O4—H4D···O3^v^	0.85	1.87	2.712 (8)	171
O5—H5D···S2^ii^	0.85	2.42	3.197 (8)	152

Symmetry codes: (i) −*x*+1, −*y*+1, −*z*+1; (ii) −*x*+2, −*y*, −*z*+2; (iii) *x*, *y*−1, *z*+1; (iv) *x*, *y*+1, *z*−1; (v) −*x*+2, −*y*+1, −*z*+1.
